# Structured education can improve primary-care management of headache: the first empirical evidence, from a controlled interventional study

**DOI:** 10.1186/s10194-016-0613-1

**Published:** 2016-03-11

**Authors:** Mark Braschinsky, Sulev Haldre, Mart Kals, Anna Iofik, Ave Kivisild, Jaanus Korjas, Silvia Koljal, Zaza Katsarava, Timothy J. Steiner

**Affiliations:** Estonian Headache Society, L Puusepa str 8H, Tartu, Estonia; Clinic of Neurology, University of Tartu, L Puusepa str 8H, Tartu, Estonia; Estonian Genome Centre, University of Tartu, Riia 23b, Tartu, Estonia; Faculty of Medicine, University of Tartu, Ravila 19, Tartu, Estonia; Department of Neurology, University of Duisburg-Essen, Essen, Germany; Department of Neuroscience, Norwegian University of Science and Technology, Edvard Griegs Gate, Trondheim, NO-7489 Norway; Division of Brain Sciences, Imperial College London, London, UK

**Keywords:** Education, Effect measurement, Headache disorders, Management, Primary care, Global Campaign against Headache

## Abstract

**Background:**

Headache disorders are under-recognized and under-diagnosed. A principal factor in their suboptimal management is lack of headache-related training among health-care providers, especially in primary care. In Estonia, general practitioners (GPs) refer many headache patients to neurological specialist services, mostly unnecessarily. GPs request “diagnostic” investigations, which are usually unhelpful and therefore wasteful. GP-made headache diagnoses are often arcane and non-specific, and treatments based on these are inappropriate.

The aim of this study was to develop, implement and test an educational model intended to improve headache-related primary health care in Estonia.

**Methods:**

This was a controlled study consisting of baseline observation, intervention and follow-up observation using the same measures of effect. It involved six GPs in Põlva and the surrounding region in Southern Estonia, together with their future patients presenting consecutively with headache as their main complaint, all with their consent. The primary outcome measure was referral rate (RR) to neurological specialist services. Secondary measures included number of GP-requested investigations, GP-made headache diagnoses and how these conformed to standard terminology (ICD-10), and GP-recommended or initiated treatments.

**Results:**

RR at baseline (*n* = 490) was 39.5 %, falling to 34.7 % in the post-intervention group (*n* = 295) (overall reduction 4.8 %; *p* = 0.21). In the large subgroup of patients (88 %) for whom GPs made clearly headache-related ICD-10 diagnoses, RR fell by one fifth (from 40 to 32 %; *p* = 0.08), but the only diagnosis-related RR that showed a statistically significant reduction was (pericranial) myalgia (19 to 3 %; *p* = 0.03). There was a significant increase towards use of more specific diagnoses. Use of investigations in diagnosing headache reduced from 26 to 4 % (*p* < 0.0001). Initiation of treatment by GPs increased from 58 to 81 % (*p* < 0.0001).

**Conclusions:**

These were modest changes in GPs’ entrenched behaviour. Nevertheless they were empirical evidence that GPs’ practice in the field of headache could be improved by structured education. Furthermore, the changes were likely to be cost-saving. To our knowledge this study is the first to produce such evidence.

## Background

Throughout Europe, and worldwide, headache disorders are highly prevalent and commonly disabling, causing heavy personal burdens and very substantial socioeconomic cost [1]. In the European Union (EU), headache disorders cost national economies well in excess of €100 billion annually [2]. Globally, headache disorders are the third cause of disability [3, 4]. Despite these compelling statistics, headache disorders are under-recognized, under-diagnosed and undertreated everywhere [1]. A principal factor in suboptimal treatment is lack of knowledge amongst health-care providers of the nature and good management of headache disorders, and this is especially so in primary care where most headache should be managed [1]. This is itself the result of very limited and wholly inadequate commitment to these disorders in medical undergraduate curricula and continuing medical education [1].

One consequence is poor outcomes. These lead in turn to patient dissatisfaction, diminished expectation, low consultation rates and persistence of these burdens largely unmitigated. Other consequences are unnecessary investigations and referrals to specialist care [1], which are wasteful of scarce health-care resources.

This spectrum of problems is encountered in Estonia, a small country of approximately 1.3 million people. Here, as elsewhere, there is a clear need for improving headache-related primary health care. Estonia is a member of the EU, but the legacy of the former Soviet health system is entrenched: purposeless over-investigation, application of arcane diagnoses, with prescription of inappropriate (often vasoactive) treatments based upon these, and very high dependence on specialist referral [5]. These behaviours are not likely easily to be changed, but the best means of doing so, with expectation of improvement in care, lies in education of health-care providers, especially in primary care [1, 6].

The aim of this study, undertaken as a project within the Global Campaign against Headache, was to develop, implement and test an educational model intended to improve headache care delivered by general practitioners (GPs) in Estonia. The primary hypothesis was that a well-structured but limited (as opposed to intensive) programme of education of GPs in headache disorders and their management would reduce referrals. We selected referral rate (RR) to specialist care as the principal outcome measure because such referrals are easily and objectively measurable. Whilst we could not, on an individual level, determine whether or not a particular referral was appropriate, we could take an informed view on whether overall RR was excessive and therefore wasteful [7]. A secondary hypothesis was that education would lessen the plethora of investigations performed by GPs in headache patients; as an outcome measure, investigation rate had the same characteristics as RR.

## Methods

### Ethics

This study was approved by the Ethics Review Committee on Human Research of the University of Tartu. Informed consent was obtained from all participants: GPs and their patients. Data-protection legislation was complied with.

### Study design

This was a controlled study, which consisted of baseline observation, intervention and outcome observation using the same measures.

### Setting and subjects

The project was conducted in the town of Põlva and the surrounding region in Southern Estonia, with a population of about 30,500. Approximately 12,500 inhabitants were registered at the only outpatients’ clinic in Põlva, where seven GPs were working. GPs seeking specialist care referred their headache patients first to the single neurologist at Põlva Hospital (level two in the European model of organization of headache services [7] described by *Lifting The Burden* [LTB] and the European Headache Federation [EHF]) (summarised in Table 1). In case of need, GPs directly and the local neurologist might both send patients further, to Tartu University Hospital, for higher-level neurological consultation (level three [7]).

**Table 1:** Headache services organised on three levels [7]

Level 1. General primary care	• Frontline headache services (accessible first contact for most people with headache)
	• Ambulatory care delivered by primary health-care providers
	• Referring when necessary, and acting as gatekeeper, to:
Level 2. Special-interest headache care	• Ambulatory care delivered by physicians with a special interest in headache
	• Referring when necessary to:
Level 3. Headache specialist centres	• Advanced multidisciplinary care delivered by headache specialists in hospital-based centres

The intervention involved the GPs and their future patients presenting consecutively with headache as their main complaint. The GPs were the research subjects. Their future patients were not subjects directly, but were beneficiaries of the intervention, who provided data for some of the outcome measures. Additionally, the records of consecutive similar past patients (who were not excluded from becoming future patients) were scrutinised to establish baseline performance of GPs.

Age, gender and educational level of each patient were registered.

### Intervention

The objective of the intervention was to provide GPs with sufficient understanding to manage, competently but not expertly, those headache disorders that are common in primary care. The intervention consisted of:

- two educational one-day (6-h) courses to all participating GPs:
  day one: didactic lectures by headache-specialists, based on the European principles of diagnosis and management of common headache disorders [8], including the recognition of important secondary headaches, and other materials in *Aids for management of common headache disorders in primary care* published jointly by LTB and EHF [9];
  day two (four weeks after day one): clarifying and reinforcing discussions between the GPs and headache specialists, including analysis of clinical cases presented by the specialists and GPs from their own practices;
- educational materials and management aids for GPs [9] translated into Estonian and (in the case only of information leaflets on headache disorders for patients) also into Russian.

### Outcome measures

Outcome measures were collected from the two groups: baseline data were collected retrospectively from past patients for the period prior to study commencement and post-intervention data were gathered prospectively from patients consulting after the intervention.

The primary outcome measure was RR: the percentage of patients referred to levels two or three. The primary analysis was the change in RR post-intervention.

Secondary outcome measures included GP-requested investigations (laboratory tests and xrays, but not CT [this and MRI are not ordered by GPs in Estonia]), GP-made headache diagnoses and GP-recommended and/or initiated treatments. Patients’ comorbidities were recorded.

All these data were acquired from the electronic medical records.

In addition, past patients as well as those included prospectively were contacted during the course of the study by telephone; those who consented participated in the following enquiries in their native language (Estonian or Russian):

- satisfaction with care, rated on a numerical rating scale (NRS) of 0–10 where 0 = “very dissatisfied” and 10 = “very satisfied”;
- adequacy of care, assessed by the Headache Under-Response to Treatment (HURT) questionnaire [10];
- burden of headache, assessed by the Headache-Attributed Lost Time (HALT) questionnaire [11];
- quality of life, assessed by the RAND 36-Item Health Survey 1.0 questionnaire (RAND-36) [12];
- health satisfaction (HS) and quality of life satisfaction (QoLS) assessed by the first two questions of WHOQoL-8 [13], rated on a NRS of 1–5 where 1 was least, 3 was neutral and 5 was the highest level of satisfaction.

### Statistics

Power calculation estimated that 273 participants were required per group assuming that RR would be 25 % at baseline and fall to 15 % after intervention and using a one-sided test with 5 % significance level and 90 % power.

Data were collected and analysed using software R, version 3.0.3 for Windows. Results were adjusted for age and gender. Means ± standard deviations (SDs) were used as descriptive statistics. Pearson’s chi-squared test was used to compare RRs between baseline and post-intervention groups, and the same and Fisher’s exact test were used for other dichotomous variables. The relationships between NRS, QoLS and HS were tested with Spearman’s correlation coefficient (CC). Student’s t-test was used to evaluate differences in RAND-36. Results were considered to be statistically significant when *p* < 0.05.

## Results

Six of the seven GPs consented to participate in the study. It became evident that our assumption regarding the baseline RR was incorrect (see below): in order to achieve adequate statistical power, the baseline group included 490 patients seen during two years prior to the first day of the intervention while the post-intervention group consisted of 295 consecutive patients consulting during one year after the second day of the intervention. There was female predominance in both groups: 73 % (357/490) in the baseline and 75 % (221/295) in the post-intervention group (the difference being insignificant: *p* = 0.58). The mean age of the baseline group (43.2 ± 15.8 years) was 3.6 years lower than that of the post-intervention group (46.8 ± 17.1; *p* = 0.004). There was no apparent difference between groups in level of education (*p* = 0.48), but over two thirds of patients chose not to disclose this information.

For the primary outcome, 476/490 baseline records (data-missing rate 2.9 %) and 294/295 post-intervention records (data-missing rate 0.3 %) were included. Baseline RR was 39.5 %; with our patient numbers we had 88 % power to see a reduction of 10 % (*ie*, to 29.5 %). However the overall reduction was only 4.8 % to 34.7 %, which did not reach significance (*p* = 0.21). When RR was analysed for different diagnoses used by the GPs according to the International Classification of Diseases (ICD-10) [14], there were clear trends towards reduction (Table 2). The most frequently used ICD-10 diagnoses at baseline were, in order, G44 (other headache syndromes), G44.2 (tension-type headache), M79.1 (myalgia [implying pericranial myalgia]), R51 (headache), G43 (migraine) and G43.9 (unspecified migraine). RR for patients with a diagnosis of tension-type headache decreased from 49 to 43 %, for patients with migraine from 46 to 33 % and for patients with non-specific headache diagnoses (G44 or R51) from 44 to 26 %. All these trends remained statistically insignificant, although the last was close (*p* = 0.06). The only diagnosis-related RR that showed a statistically significant reduction was that for (pericranial) myalgia (19 to 3 %; *p* = 0.03).

**Table 2:** Referral rates according to ICD-10 diagnoses before and after intervention

ICD-10 diagnosis	Referral rate		*p*
	Baseline	Post-intervention	
G44.2 Tension-type headache	58/119 (49 %)	50/115 (43 %)	0.50
G43 Migraine	22/48 (46 %)	15/45 (33 %)	0.31
G44 Other headache syndromes and R51 Headache	79/178 (44 %)	10/38 (26 %)	0.06
M79.1 (Pericranial) myalgia	20/108 (19 %)	1/36 (3 %)	0.03
Total for four headache diagnostic groups	179/453 (40 %)	76/234 (32 %)	0.08

**Fig. 1:**
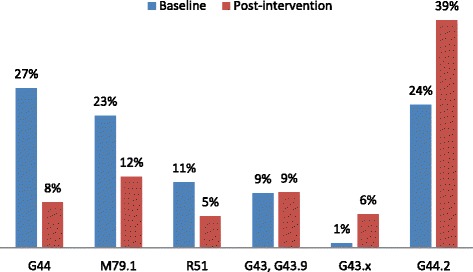
Usage of the most frequent diagnoses before and after intervention. G44: other headache syndromes; M79.1: (pericranial) myalgia; R51: headache; G43: migraine; G43.9: unspecified migraine; G43.x: specified migraine subtype; G44.2: tension-type headache. The figure depicts a clear trend, post-intervention, away from use of non-specific diagnoses towards more specific diagnoses

Within the spectrum of headache diagnoses used by GPs, there was a significant increase towards using those that were more specific (Table 3, Fig. 1).

**Table 3:** Usage of the most frequent diagnoses before and after intervention

ICD-10 diagnosis	Baseline	Post-intervention	*p*
G44 Other headache syndromes	134 (27.3 %)	23 (7.8 %)	<0.0001
G44.2 Tension-type headache	120 (24.5 %)	115 (39.0 %)	<0.0001
M79.1 (Pericranial) myalgia	111 (22.7 %)	36 (12.2 %)	0.0003
R51 Headache	55 (11.2 %)	16 (5.4 %)	0.009
G43 Migraine and G43.9 Unspecified migraine	46 (9.4 %)	28 (9.5 %)	1
G43.0, G43.1, G43.2 and G43.8 Specified migraine subtypes	4 (0.8 %)	17 (5.8 %)	<0.0001

**Fig. 2:**
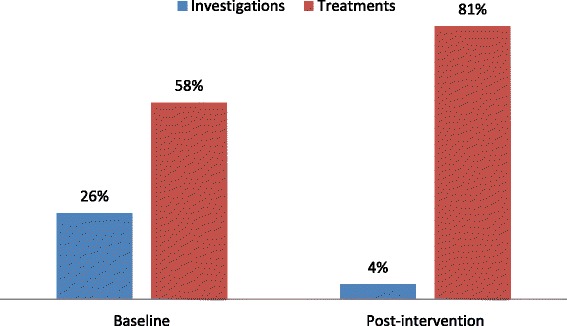
Requests for investigations and initiation of treatment before and after intervention. The figure depicts a reduction in the former and an increase in the latter post-intervention

Requests for diagnostic investigations for headache reduced from 26 % (125/490) at baseline to 4 % (13/295) post-intervention (*p* < 0.0001) (Fig. 2). Within these numbers, laboratory investigations fell from 22 to 1 % (*p* < 0.0001) and xrays from 7 to 3 % (*p* = 0.038). These changes were noted within all diagnostic groups, being most clear-cut for migraine, for which there were no investigations performed at all post-intervention (reduction from 14 to 0 %; *p* = 0.01).

Initiation of treatment by GPs increased from 58 % (286/490) at baseline to 81 % (239/295) post-intervention (*p* < 0.0001) (Fig. 2). Furthermore, use of more than one treatment option increased from 14 % (67/490) to 41 % (120/295) (*p* < 0.0001).

Proportions responding to enquiries into satisfaction with care were low: 155 patients (31.6 %) at baseline and 87 (29.5 %) post-intervention. Non-responders in the baseline group were somewhat younger (41.9 ± 15.8 *versus* 46.1 ± 15.2 years; *p* = 0.006) and included more men (31 % *versus* 19 %; *p* = 0.04). Overall satisfaction with care showed very little change in NRS mean score: from 6.4 ± 2.5 at baseline to 6.7 ± 2.3 post-intervention, which was not significant (*p* = 0.36). We found no differences by diagnostic groups, or in those in whom investigations were performed or treatments initiated, or in those referred.

There were, similarly, no significant changes in HS (*p* = 0.22) or QoLS (*p* = 0.66). The number of comorbidities was strongly correlated with lower scores in HS in both groups (baseline CC: −0.23; *p* = 0.004; post-intervention CC: −0.32; *p* = 0.002). Proportions responding to the HALT and HURT questionnaires were also low: 34.9 % in the baseline and 29.8 % in the post-intervention groups. Again, non-responders at baseline included more men (32 % *versus* 19 %; *p* = 0.003). There was no meaningful change between groups in either HURT or HALT scores (mean HALT scores were 2.8 (±1.3) at baseline and 2.9 (±1.3) post-intervention; *p* = 0.49). There were no significant overall changes in QoL measured by RAND-36, or in any of the domains.

## Discussion

This controlled intervention study has shown that GPs’ behaviours were changed and practice improved by a structured educational programme, albeit not in a way that was reflected in all measures. In particular, the fall in RR (the primary outcome measure) was statistically insignificant; on the other hand, GPs made more disease-specific diagnoses while requesting far fewer investigations, and they became much more willing to initiate treatment. It should be noted that, despite the difference in their sizes, the two groups of patients on whom these comparisons were based were demographically similar. Although the age difference (43.2 *versus* 46.8 years) was significant statistically, it was not so clinically.

To our knowledge this is the first study to demonstrate empirically that GPs’ practice in the field of headache can be favourably influenced by education. The Dutch study of Smelt et al. [15], aimed specifically at migraine management and recruiting patients already on triptan therapy, failed to show a beneficial effect. This controlled trial employed clinical outcomes, and perhaps demonstrated the difficulties associated with them. The Norwegian study of Kristoffersen et al. [16], targeting medication-overuse headache only, improved outcomes in an intervention group but the essential element of the intervention was to equip GPs with a “simple and effective instrument” as a management aid; the educational element was secondary to this. Both these studies had much narrower focus than ours; we assessed the provision of headache care to unselected patients, which was a major strength in both purpose and study design.

That there was only a small and statistically insignificant reduction in the primary outcome measure – the overall RR – was disappointing, especially since baseline RR was much higher (39 %) than anticipated (25 %). However, for patients with clearly headache-related GP-made diagnoses, RR fell by one fifth (from 40 to 32 %; *p* = 0.08), suggesting some gain in confidence in managing patients with primary headache without referral to a neurologist.

Only for patients diagnosed with (pericranial) myalgia was RR reduced significantly, but this outcome was substantially influenced by a considerable reduction in use of this diagnosis (M79.1). We did not analyse diagnostic changes case-by-case or perform quality analysis of diagnoses, but can reasonably speculate that, with better knowledge applied to recognizing and diagnosing tension-type headache, inappropriate use of M79.1 gave way to correct usage of G44.2 for this disorder. In keeping with this supposition, employment of the latter diagnosis significantly increased post-intervention. Also there was a significant reduction in diagnoses that did not specify a primary headache type. In other words, patients left their GPs’ offices less frequently with diagnoses of headache as a symptom and more frequently with diagnoses naming the disease causing this symptom. GPs’ practice shifted towards themselves diagnosing the most common primary headaches.

Key to this is that primary headaches are diagnosed clinically, rarely with need for investigations. Our results show that, post-intervention, GPs substantially reduced their demands for diagnostic investigations, eliminating them altogether in the case of migraine. Not only was this further evidence of learning and of confidence gained as a result, but also it produced immediate cost savings. Our study was not designed to measure cost-effectiveness, but this finding may be one of particular importance because it indicates that health-care resources can be conserved.

In line with an increased rate of specific diagnosis, the study found a greatly enhanced rate of GP-initiated treatment. At baseline, 42 % of patients appeared to receive no treatment recommendations from their GPs; post-intervention, this fell to 19 % – that is to say, 81 % of patients left their GPs’ offices with clear treatment options recommended or prescribed. It was not possible in the context of the study to make any judgement of the appropriateness of treatment initiated.

Interestingly, all these changes in GPs’ practice post-intervention had no discernible influence on patient-reported outcomes – satisfaction with care, satisfaction with health and quality of life, lost productive time or quality-of-life measures. It should be noted here that the instruments used, while not directly validated in the study population, had all been employed in multiple countries, cultures and languages. Where patient satisfaction is concerned, there are multiple determinants that might explain failure to indicate benefit. For example, we sought to reduce the use of investigations because these do not contribute usefully to the diagnosis of headache disorders in primary care. Similarly we aimed to reduce RR because the proportion of patients who should be referred – for diagnostic or management difficulties or for secondary headache – is much smaller than was the baseline RR [7]. Patients, however, might not agree that these reductions were in their interest. Also we have to recognise that the study was underpowered at outset for these secondary outcome measures, added to which the proportions responding to the patient-directed enquiries were about 30 %. Realistically we should not attempt to make anything of these, because, with such low response rates, bias was also likely. Unfortunately, such response rates are not at all unusual [2] and, as was the case here, are one of the limitations of this type of study.

Other limitations here related to the scope of the study. Its aims did not include quality review of diagnoses, investigations or treatments; to do any of these would have required an entirely different approach. Also this study cannot comment on the duration of found effects, which was outside the scope of the protocol. In other words we are not able to answer the question about need for repeated interventions over time. Further studies, which are planned, are required for these purposes.

Finally it must be said that the gains achieved, at least in the principal outcome measure, were less than had been hoped for, but the fact that gains were made suggests more would be achieved with more educational input. The cost-effectiveness of this must be investigated, but is very likely to be favourable given the enormously high socioeconomic burden of headache [1, 2].

## Conclusions

GPs’ practice in the field of headache can be improved by a structured yet limited educational programme. To our knowledge this study is the first to show this empirically. Improvements were modest: established practice is entrenched and change is likely to be slow. But the improvements included wider use of headache diagnoses employing accepted and more specific terminology, less demand for investigations, which are almost invariably unhelpful, less dependence on referral, which is often unnecessary, and greater willingness to initiate treatment. These changes can be expected to lead to immediate cost savings.
